# How Does Green Innovation Strategy Influence Corporate Financing? Corporate Social Responsibility and Gender Diversity Play a Moderating Role

**DOI:** 10.3390/ijerph19148724

**Published:** 2022-07-18

**Authors:** Sohail Ahmad Javeed, Boon Heng Teh, Tze San Ong, Lee Lee Chong, Mohd Fairuz Bin Abd Rahim, Rashid Latief

**Affiliations:** 1 School of Management, Hunan City University, Yiyang 410215, China; sohailahmaduaf@yahoo.com; 2 Faculty of Management, Multimedia University, Persiaran Multimedia, Cyberjaya 63100, Selangor, Malaysia; llchong@mmu.edu.my (L.L.C.); fairuz.rahim@mmu.edu.my (M.F.B.A.R.); 3 School of Business and Economics, Universiti Putra Malaysia, Serdang 43400, Selangor, Malaysia; 4 Department of Business and Administration, Daffodil International University, Daffodil Smart City, Dhaka 1207, Bangladesh; 5 School of Finance, Xuzhou University of Technology, Xuzhou 221000, China; rashidlatief@xzit.edu.cn

**Keywords:** green innovation strategy, corporate financing, corporate social responsibility, gender diversity, environmental pollution

## Abstract

Global warming is becoming more and more of a concern, leading authorities to take action. The industrial sector is a key contributor to environmental and social problems. Based on stakeholder theory and agency theory, this research proposes that green innovation strategies at the firm level can overcome the industry’s negative environmental impact. As a result, the focus of this research is on green innovation strategies for corporate financing. In addition, this research suggests that corporate social responsibility and gender diversity directly affect corporate financing and their interaction. This study used Chinese 301 manufacturing firms (3010 observations) for the period 2010–2019 for this purpose. This study looks into panel data issues in depth by using approaches such as the fixed effect and generalized method of moment. The feasible generalized least square was employed to increase robustness. Furthermore, green innovation strategies were used for corporate financing. Second, the study discovered that corporate social responsibility aided firm financing. Our findings also imply that corporate social responsibility helps to attenuate the association amid green innovative strategies and corporate financing. Finally, these findings revealed that gender diversity had a favorable effect on corporate financing. Furthermore, this study confirmed that the moderating role of gender diversity is beneficial to green innovative strategies and corporate financing. These findings add to the literature by providing policymakers and regulatory bodies with useful information for advancing sustainable development.

## 1. Introduction

The issue of global warming has been highlighted by various scholars these days. Following the continual deterioration of the natural atmosphere, voices advocating for environmental safety have become progressively loud in recent years [1]. Many countries have made significant efforts to achieve green growth since the Kyoto Protocol in 1997 and the Paris Agreement in 2016 [2]. Moreover, organizations and governments are putting a lot of work into addressing global warming and other societal challenges that are becoming more prevalent by the day [3]. To tackle global warming, several countries have developed environmental strategies, which may be characterized as a set of rules that organizations or governments can utilize directly or indirectly to solve environmental challenges [4]. Before this, a number of academics stated that the corporate sector significantly contributes to global warming and other environmental problems [5,6]. While the industrial sector helps a country thrive economically, it negatively influences the environment [7]. While China’s economic Reform and Openness has accelerated, environmental contamination has become a big glitch.

Liu et al. [8] assert that China released 2.49 rigatonis of carbon dioxide (CO_2_) from fossil fuels in 2013, resulting in a slew of harmful consequences for the country’s well-being and reputation. Auspiciously, China’s government has established several regulations to address the dire situation, particularly in the areas of industry and entrepreneurship, which play a critical role in pollution and emissions. For example, to encourage business environmental responsibility, programs such as green insurance, green credit, and green security have been developed [9]. Moreover, this research has a significant impact on the context of emerging economies because environmental worries are very important in emerging countries, and the industrial sector is not very established in developing countries [10]. Businesses in developing nations do not have the financial resources to invest in environmentally friendly practices in the same way that businesses in wealthy ones do [11].

In this context, green innovation methods have been more popular at the corporate level due to the perception that the industrial sector considerably contributes to environmental and social concerns [12]. Green innovation techniques play an important role in reducing the harmful effects of industry [13]. Based on this concept, every stakeholder is encouraged and pressured to carry the responsibility for long-term economic growth as a result of such policies [14,15]. In this setting, many green innovations have led to strategic activity for Chinese enterprises which look to improve their environmental practices [16,17]. In addition, several scholars also claimed that green innovative strategies may enhance company financing activities [12,13,18]. Moreover, Abbas and Sağsan [19], as well as Albort Morant, Leal Millán, and Cepeda Carrión [14], define green innovation as “developments and renewals done to regulate discharges, diminish pollution, and save money”.

Likewise, enterprises in China, such as those in other emerging countries, are facing more financing restrictions and anxieties associated with finances or capital [20]. As stakeholders become more concerned about the social and environmental glitches, company green innovation may play a critical role in alleviating financing restrictions [7]. Furthermore, a firm with better corporate financing can enhance profit as well as participate in environmental practices. As a result, the initial goal of this research is to see if green innovation can help businesses overcome their financial restraints.

For supporting the first objective of this study, the agency theory explains the link between green initiatives and business financing [21,22]. Previous research by Li et al. [23] and Passetti et al. [24] has demonstrated that company environmental confession can minimize information asymmetry and maximize ways of corporate financing. The preceding work acknowledged the reputation of green innovative strategies for corporate financing [12,13,18]; however, no study has addressed the elements that contribute to a favorable association. What are the essential factors that lead to this advantageous association if green innovative strategies can increase business financing?

The present responses to the aforementioned questions have clear gaps. Consequently, this study advises that the concepts of CSR and gender diversity can be used to improve green innovation strategies and corporate financing because a company’s commitment to green innovative practices is influenced by corporate social responsibility [25]. In this aspect, several earlier studies, such as those by Dhaliwal et al. [26] and Cheng et al. [27], have verified the positive association between corporate social responsibility (CSR) and corporate financing. In addition, CSR may assist a company, not just in dealing with environmental challenges, public needs, and social growth, but also in making better financial decisions [28]. Moreover, transparency in CSR performance influences financial decisions by lowering capital limits. Firms that prioritize CSR practices can improve their potential to develop new green practices [29,30]. Furthermore, one of the most essential elements for stakeholders in a company’s CSR initiative is to improve the green innovation [31]. In this regard, Tomomi and Management [32] point out that CSR promotes company competitiveness and is likely to lead to green innovation. Furthermore, Al-Abdin et al. [33] and Madueno et al. [34] stated that CSR has a significant impact on economic and environmental performance for green innovation in developing nations. In addition, Albino et al. [35] highlighted that CSR is one of the most important tools for promoting green innovation that fulfils consumer needs.

Furthermore, this study reveals that gender diversity has a significant impact on corporate finance and green innovation activities. For example, gender diversity improves corporate funding for societal activities in the same way that it improves the firm’s long-term profitability [36]. Moreover, women are more concerned with social practices than men [37] because females are constantly looking for a positive long-term reputation, which drives them to promote social events that benefit all parties involved [38]. In addition, the agency theory stated that a board with more diversity is more likely to be a better monitor of managers’ performance since diversity improves financing decisions [39]. Harjoto and Rossi [36] also reported that female directors encourage businesses by employing green innovative strategies to resolve agency concerns. Many experts believe that gender diversity is vital to improve green innovation operations and corporate finance [39]. Therefore, this study has a major motive to investigate the influence of corporate social responsibility and gender diversity as moderators on the link between green innovation strategies and corporate financing. As far as we know, no previous study or scholar has investigated the role of these variables in green innovative strategies and business finance. For the completion of the above-mentioned objectives, this study incorporates several econometric techniques, such as fixed effects to manage unobservable heterogeneity, the generalized method of moment (GMM) model to tackle endogeneity difficulties, and a feasible generalized least square (FGLS) as a robustness test. This work contributes to the following five areas. The first finding of this study states that green innovative strategies are positively linked to corporate financing. Moreover, this study observes the direct effect of CSR and gender diversity on corporate financing before utilizing them as moderators. Therefore, the second finding discovers that CSR has a favorable impact on corporate financing. Thirdly, the work demonstrates that CSR not only enhances corporate finance, but also acts as a moderator in the affiliation amid green innovation strategies and corporate financing. Fourthly, gender diversity and corporate financing have a favorable association, which reveals that gender diversity serves as a moderating factor in establishing a positive link between green innovative strategies and corporate financing.

Finally, our research adds to the literature by serving as a guide for policymakers to improve firm-level sustainability. Regulatory bodies can focus on this tractor to eliminate negative industrial results. In addition to this, these findings support the inclusion of gender diversity and corporate social responsibility initiatives to accomplish long-term sustainable goals.

**Figure 1:**
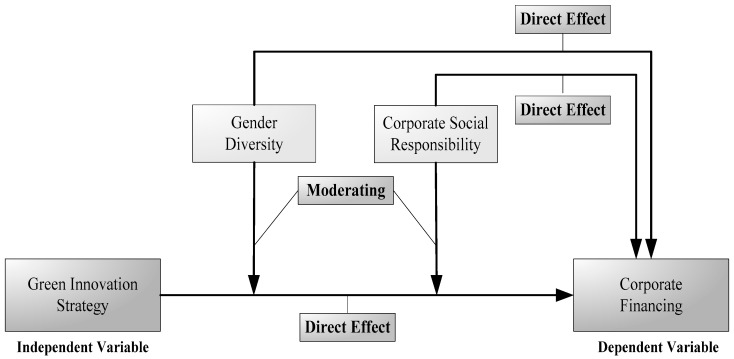
Conceptual roadmap.

The study’s leftovers are divided into several areas. The theoretical analysis and empirical discussion for hypothesis construction are explained in Section 2. Data collection, variable measurement, and research methodologies were discussed in Section 3. Section 4 offers the results and comments. The study’s findings, ramifications, limitations, and future directions are summarized in Section 5. Figure 1 depicts the study’s conceptual framework.

## 2. Hypothesis Construction and Theoretical Discussion

### 2.1. Theoretical Examination

The stakeholder concept is a good place to start when looking at green innovation. The government, corporate management and employees, creditors, shareholders, and financial institutions are examples of corporate stakeholders [21,40]. Stakeholder theory states that enterprises and stakeholders can obtain the resources needed for development and provide a positive connection between them [41]. Green innovation can assist companies that not only handle environmental glitches, but also those which address the demands of the public and encourage social growth. Furthermore, it has the potential to boost the company’s image [28]. A company’s reputation is its most basic intangible asset. A company’s social reputation can be improved by enhancing its relationships with external stakeholders [42]. This reputation can then be used to recruit better personnel or to boost corporate financing, loyalty, and devotion to the company [28].

Corporate social practices and media praise are all examples of how reputation may help a company obtain capital support from multiple stakeholders [43,44]. Stakeholder theory also offers a positive link between CSR and performance, in which CSR engagement leads to improved stakeholder affairs, which decreases an organization’s transaction expenses [21,45]. Corporate social practices improve a company’s reputation and increase shareholder confidence, which boosts corporate funding [21]. As a result, the stakeholder theory promoted corporate social responsibility as a factor in corporate funding. On the other side, Jensen and Meckling [22] also proposed the agency theory as a theoretical basis for relating CSR to corporate financing. The agency hypothesis predicts that when information asymmetry exists, managers can make a series of decisions to maximize their interests. CSR disclosures may serve as a monitoring tool in this environment by minimizing information asymmetry and agency issues [46].

The role of CSR and gender diversity as a moderator on the link between green innovation strategies and corporate finance is supported by both stakeholder and agency theories [21,22]. Prior research suggests that organizations that voluntarily implement CSR policies exhibit less opportunistic actions [47,48] and higher stakeholder involvement than firms that do not embrace CSR policies [27]. In this situation, CSR disclosures are intended to boost trust between the company and its stakeholders, notably its lenders. The latter may incentivize this by giving companies with high CSR disclosure scores which provide easier access to financing. Additionally, this theory backs the inclusion of gender diversity because, from an agency perspective, gender diversity encourages stronger control of managers’ behavior by improving corporate financing [39,49]. As a result, the presence of female participation on the board may have an impact on how the board’s features stimulate the company’s capital arrangement. Moreover, board members may be chosen based on their gender diversity, which may have an impact on funding decisions. Finally, the influence on the firm’s capital structure is stronger when the board has the same ratio of gender.

### 2.2. Hypothesis Development

#### 2.2.1. Green Innovation Strategy and Corporate Financing

The notion of green innovation is included in many stages of technical innovation, resulting in a reduction in material consumption and environmental effects on the protection of the mind [50]. The creation of new or better processes, practices, institutions, and goods that enhance the environment and maintain its long-term viability is referred to as environmental innovation [51]. It also refers to a collection of manufacturing techniques that include pollution prevention, emission reduction, and conservational management arrangements [52]. A variety of methods and production procedures that diminish negative environmental influences is also part of a green innovation strategy, which also consider the use of non-toxic materials. During the product’s creation, it was found to be nontoxic and easily disassembled.

Governments are more likely to assist legitimate businesses [53]. Green innovation can reveal whether or not a company is legal and operating efficiently. If a company’s legitimacy is called into question, the public will denounce and attack it; thus, the monitoring authority will tighten its oversight. The business’s ability to access a variety of resources will also be harmed. Furthermore, society will recognize and value green innovation strategy as a manifestation of ethics. Stakeholders will view the company to be relevant and appealing, and will argue that the company’s characteristics are comparable to those of the shareholders by providing the firm with a greater level of gratitude [54]. Because green innovation influences stakeholders’ perceptions of the organization, these practices will help to foster trust and goodwill. Stakeholders will now actively assist the company’s environmental practices by providing energetic resource funding. As a consequence of the firm’s outstanding environmental performance, stakeholders’ financial backing for the company will instantly increase [13].

Previous research has not looked into the link between green innovation strategy and corporate financing. Because every business’s purpose is to maximize profits, corporations will approach environmental innovation largely from an economic stance [30]. Green innovation allows businesses to attract additional investors, creditors, suppliers, and other stakeholders, as well as gain more resources [13]. It will be difficult for a company to survive and develop if it cannot establish positive relationships with its stakeholders. The green innovation approach has a strong philanthropic drive and emphases decreasing environmental contamination and energy feasting in the manufacturing course. As a result, it enhances business financing and contributes to a win–win scenario for the company and its stakeholders [3]. Previous research on the relationship between green innovation strategy and financial success has yielded conflicting consequences [55]. According to Grewatsch and Kleindienst [12], 59% of studies found a favorable association between a firm’s green operations and profitability, while 41% of scholars found diverse or negligible consequences.

According to the literature, all environmental difficulties may mend a company’s competitive situation by lowering manufacturing costs and increasing amenability [56,57,58]. Green innovation strategy installation that boosts efficiency, generates a competitive gain, and creates the company’s status has a good impression on financial practices [55,59,60]. These elements immediately improve corporate finance. Furthermore, the green firms’ reputation aids in enhancing their ability to obtain money from any financial institution, consistently lowering the firm’s risk and stabilizing cash flows [61]. Sharfman and Fernando [62] make similar arguments, claiming that reducing pollution and the use of hazardous substances reduces litigation risk, enhances corporate financing, and lowers risk. Generally, in financing, the influence of green innovation strategy on company finance may be achieved through several stakeholders, including original and potential shareholders [63]. Based on these reasons, we have postulated the following hypothesis.

**Hypothesis** **1** **(H1).** *Green Innovation Strategy Can Help for Raising Corporate Financing*.

#### 2.2.2. The Role of Corporate Social Responsibility

Since Friedman’s new classic vision of CSR was first presented in 1970, the concept of CSR has been at the centre of financial, economic, and political arguments. In business decision-making and conduct, CSR echoes a company’s attention to moral and ethical issues [28]. CSR’s genuine impact goes beyond these issues by instilling a sense of trust and belonging across a wide range of stakeholders. According to an empirical study, CSR practices greatly improve the information environment [26]. In addition, Bae et al. [64] reported that CSR performance is highly important for corporate financing. Other researchers have also claimed that CSR can benefit society by improving access to corporate financing [65]. Furthermore, Cheng et al. [66] demonstrate the mechanisms by which increased CSR performance leads to fewer financing restrictions.

Additionally, Sharfman and Fernando [62] discover that firms with proper CSR performance can improve corporate financing in the long run. Similarly, Hamrouni et al. [67] have investigated the link between CSR performance and corporate financing in French firms, and their findings also endorse the role of CSR in the improvement of corporate financing. In terms of capital structure, Cheng, Yang, and Sheu [27], among others, show that CSR performance transparency influences financial decisions by decreasing capital limitations. Indeed, Increased CSR information availability and quality minimize knowledge asymmetry between a company and its investors [68,69], resulting in lower equity costs [26,70] and capital constraints [68].

According to Lins et al. [71], companies with high CSR ratings have more profitability, growth, and sales per employee than companies with poor CSR ratings and they also take on more financing. Erragragui and Finance [72] use a panel of American enterprises to determine that environmental and governance capabilities cut firms’ borrowing costs, as established in earlier studies. Moreover, social responsibility is a notion of action in which a company is considered as a social character with various stakeholders’ attention to achieve a specific task [73]. Even though the impact of CSR programmers has received a lot of attention in the literature, the impact of CSR on manufacturing and how CSR might assist improve competitiveness through green innovation is still largely studied [74]. Management, sustainability certification, and reporting are examples of CSR tools, which reflect defined management techniques for the advancement of green innovation, such as the application of sustainability labels [75].

CSR’s involvement in incorporating green innovation factors into corporate plans for survival and smooth operations in the ever-changing business environment has been shown by researchers [76,77]. Additionally, enterprises with green innovative practices attract more buyers since they prefer to engage with companies that practice CSR [78]. According to Wang et al. [79], adopting CSR can reduce environmental impact by minimizing industrial waste, improving recycling, and lowering manufacturing costs which will lead to firm green innovation. Previous studies have found that CSR programs have a direct and beneficial impact on the adoption of green practices, corporate sustainable performance, and corporate green performance [80,81,82]. Furthermore, when it comes to CSR and organizational performance, academics have underlined the importance of innovation [83,84]. Environmentalists have praised manufacturing companies for incorporating CSR practices and green thinking into their operations to reap the benefits of environmental and economic sustainability [85].

Boehe and Cruz [86] exposed that paying response to social practices promotes product distinctiveness and internationalization chances to marketplaces with more active green customers, enhancing market performance and business turnover over time [87]. Firms with a precise category of CSR focus can improve their potential to make green innovations [29,30]. The worth of green innovation is undeniably associated with the ability to increase environmental performance while also adhering to environmental rules. Therefore, green innovation is viewed not just as a means of addressing environmental glitches, but also as a means of fostering long-term business success [88]. Based on these reasons, we have postulated the following hypotheses.

**Hypothesis** **2** **(H2).** *Corporate social responsibility is beneficial for corporate financing*.

**Hypothesis** **3** **(H3).** *Corporate social responsibility helps to moderate the connection between green innovation strategy and corporate financing*.

#### 2.2.3. The Role of Gender Diversity

In current years, scholars and the general press have realized that workplace diversity is a problem that has gained a lot of attention [89]. Schubert [90] and Maxfield et al. [91] claim that there is more female participation in risk activities than male participation, based on the gender diversity doctrine. This means that, in the presence of women, risk decisions are lower, while risk decisions in the presence of men are higher. Above all, the link between aversion and capital arrangement has yet to be properly inspected, and it is still in its infancy, mainly in emerging nations. According to Schicks [92], male borrowers are highly over-indebted than female borrowers. One probable explanation for this outcome is that the risk aversion concept may assist women in avoiding financial dangers, causing them to use less debt.

Additionally, according to Virtanen and Governance [93], female board members actively participated in board decisions more than males. Ruigrok et al. [94] also highlighted that through their extensive impact on the decision-making process, female directors can add to board effectiveness. Boards with gender diversity are less associated with debt costs as the existence of females executives on the board reduces management opportunistic actions and information asymmetry [95]. This has an impact on lenders’ assessments of a borrower’s capacity to repay a debt with interest. Nguyen et al. [96] highlighted that enterprises with increased gender diversity may influence corporate financing decisions.

Furthermore, Jensen and Meckling [22] claimed that the amount of corporate financing increases the firm’s worth. A tax benefit for interest expenditures, in particular, stimulates financing to reduce tax expenses and raise the company’s worth. Firms with higher gender diversity are more likely to employ financing in this way, especially if they want to increase their value. One likely rationale for this idea is that debt financing is less expensive when women are on the board, which acts as a catalyst for taking on additional debt. As a result, the board’s effect will be stronger when the gender distribution of board members is even, since an even sharing of male and female board members boosts the board’s performance. Zaid et al. [97] probed gender diversity as a moderator on the link between corporate financing and corporate governance in Palestinian listed firms. Their outcomes supported the positive role of gender diversity for association amid corporate governance and corporate financing.

According to Usman et al. [98] the presence of women on the board of directors is associated with financial decisions made by the company. Moreover, Schicks [92] reported that women on the board can manage corporate financing decisions better than males. Similarly, Virtanen and Governance [93] also highlighted the importance of females for corporate financing in firms. Ruigrok, Peck, and Tacheva [94] stated that female directors can contribute to board effectiveness through their widespread influence on the decision-making process. Additionally, it has been demonstrated that organizations with gender-diverse boards have lower debt costs because the presence of women directors in the boardroom reduces managerial opportunistic behavior and knowledge asymmetry [98]. Likewise, Elmagrhi et al. [99] suggest that organizations with gender diversity representation may need to manage debt properly to counteract opportunistic behavior of managers that may stem from probable deficient management oversight, which will automatically boost corporate finance.

In addition to this, the influence of gender diversity on company social practices is important. Female directors have been linked to strong, long-term practices in several research [100,101]. Furthermore, Harjoto et al. [102] discovered that female executives are strongly associated with sustainable development compared to male executives. Female directors are also important in enhancing a company’s social activities, according to a meta-analysis study [103]. Likewise, Harjoto and Rossi [36] discovered that having female executives helps corporations in long-term sustainable company social practices.

Boukattaya and Omri [104] also emphasized the importance of female directors in enhancing environmentally sustainable operations. A number of researchers have looked into gender diversity and green practices, including [102,105]. The existence of female executives was also established as a vital component of the green innovation strategy. Moreover, Landry et al. [106] stated that having female executives on a board of directors reflects a company’s ethical behavior, which leads to a better reputation in society. Furthermore, organizations with female directors have strong corporate social programming [107]. By participating in social events, female directors attempt to capture more profit for the happiness of shareholders, and they attempt to capture more profit for the satisfaction of shareholders [38]. Based on these reasons, we have postulated subsequent hypotheses.

**Hypothesis** **4** **(H4).** *Gender diversity can also help for raising corporate financing*.

**Hypothesis** **5** **(H5).** *The link between green innovation strategy and corporate financing is positively linked to gender diversity*.

## 3. Methodology

### 3.1. Selection of Data and Samples

For this study, the Shanghai and Shenzhen stock exchanges were chosen as Chinese stock markets in the “A” category. Manufacturing companies were chosen for examination since previous research showed that they were heavily involved in environmental issues and needed to be investigated [108,109]. Moreover, Chinese manufacturing firms are thought to be more polluting [110]. According to the Environmental Protection Agency, manufacturing enterprises are a major source of waste, air, and water pollution, as well as a significant contributor to climate change [111]. As a result, manufacturing corporations are more responsible than other industries for disclosing accurate information on corporate social issues [112]. Therefore, this study chose manufacturing firms for the probe. We started by gathering patent data from the company’s annual reports, as well as vital statistics from the China Stock Market and Accounting Research database (CSMAR). The information was gathered between 2010 and 2019. Finally, 301 companies (3010 observations) were chosen to finish the inquiry.

### 3.2. Measurement of Variables

#### 3.2.1. Corporate Financing

Mulatu et al. [113] discover that the right and fair selection of variables has distinct ramifications on the results, according to corporate finance research. The ratio of long- and short-term borrowings to total assets was used as the measure in this study, with corporate financing as the dependent variable [13,114].

$$\frac{Long\text{-}term\;and\;Short\text{-}term\;Borrowings}{Total\;Assets}$$

#### 3.2.2. Green Innovation

According to a previous researcher, the proper measurement of variables yields successful outcomes in empirical studies [115]. In general, companies that invest in exclusive rights are thought to be engaged in green innovation strategies [116]. As a result, we used environmental patent applications filed by businesses as an example of green innovation [116,117]. We often chose companies that have invested in patents for green innovation, which include terms such as green, clean, sustainable, cycle, saving, ecological, environmental protection, low carbon, and decrease in greenhouse gasses and emissions [118,119]. Generally, businesses engage in patents to retain their social image and gain technological advantages, as well as to increase revenues. As a result, patent filings are thought to be the finest instrument for evaluating a company’s innovative methods [116]. As a result, we track green innovation strategies by the number of patents filed by businesses across time [120].

#### 3.2.3. Corporate Social Responsibility

Feng et al. [121] stated that the proper measurement of CSR actions has more reliable results. CSR measuring indices have been developed by several research studies, most of which address similar elements such as human rights, state rights, stakeholder rights, community rights, and others [122]. On a wide scale, a CSR index was built based on minority rights such as castes, tribes, children’s meals, and mixed marriages, as well as business annual reports with CSR disclosure. Furthermore, environmental considerations such as hazardous gases were also analyzed. Another study calculated CSR in Korea and a CSR index was created based on five indicators: staff training and education; firm charity activities; standard reporting format for accounting, auditing, and the environment; customer trust; and environmental engagement [123].

Furthermore, a researcher used Thomson Reuters ASSET4 to deliver CSR scores to corporations for societal and ecological participation [124]. Moreover, another study analyzed the implementation and consequences of CSR by measuring the societal, economic, and environmental elements of CSR by businesses [125]. On the other side, Ehsan et al. [126] reported that CSR refers to a company’s efforts that are not only concerned with profit but also with social acts. Thus, following the Shanghai Stock Exchange’s requirements, we employed a CSR index known as social contribution value per share (SCV) [121]. This index is based on all of the components required for social value, such as earnings per share, the value produced for society as assessed by state tax revenues, employee salaries, creditors’ loan interest, and other values for stakeholders, by eliminating environmental damage as a social cost. According to Feng, Chen, and Tang [121], this index covers all required aspects for corporate social practices. So, CSR is calculated as follows:

$$\frac{EarningsPer\;Share+\left(Staff\;Expenses+Total\;Taxes+Public\;Welfare\;Expenses+Interest-Social\;Cost\right)}{Total\;Equity}$$

#### 3.2.4. Gender Diversity (GD)

In this study, gender diversity is used as a moderating variable. The proportion of females on the board is used to calculate the gender diversity of the board. According to Yasser et al. [127], Hyun et al. [128], and Kassinis et al. [129], this gender diversity assessment is important and has been used in prior imperative studies. As a result, gender diversity is measured by the formula below.

$$\frac{Total\;Number\;of\;Women\;on\;Board}{Total\;Number\;of\;Board\;of\;Directors}$$

#### 3.2.5. Control Variables

This study incorporated a lot of control factors to achieve the best findings. Among all the control variables in corporate finance, the size of the company is the most important. For empirical studies in corporate finance, firm size is important, although the results differ by industry. In most corporate social studies, the size of the company has a significant and favorable impact [7]. Thus, to begin, the natural log of total assets is utilized to determine the size of the company [110]. Second, Fang et al. [130] highlighted that, for corporate finance and environmental studies, liquidity plays a role as a control variable. Roy et al. [131] also supported the role of liquidity in corporate social practices.

Therefore, to determine liquidity, the ratio of a company’s current assets to current liabilities is computed [132]. Moreover, Sayilgan et al. [133] stated that for corporate finance studies such as capital structure, the PPE ratio has a significant role. Hence, the net ratio of plant, property, and equipment is computed by dividing the total firm sales by the net of plant, property, and equipment [7]. Fourth, the asset turnover ratio as a control variable supports the empirical studies, especially on corporate financing [134]. Thus, asset turnover is calculated as the ratio of total sales to total assets [135]. Lastly, this study employed environmental awareness as a control variable for supporting the corporate social practices results [136]. Finally, environmental awareness is calculated by dividing the total number of employees by the amount of money spent by the company on landscaping and other greenery reasons [7].

### 3.3. Empirical Strategy

This study used panel data, and previous research has found that panel data are commonly associated with endogeneity difficulties [137,138]. The endogeneity problem is characterized as a relationship between the error term and the explanatory variables that leads to distorted and unreliable outcomes [7]. Endogeneity bias is also induced by imprecise inferences and inconsistent evaluations, which can result in confusing results and incorrect theoretical interpretation [139]. Despite this, the majority of academics working with panel data have not addressed the issue of endogeneity. Furthermore, 90% of published research on panel data, for example, ignores endogeneity issues [140,141].

In this regard, some academics have developed statistical strategies for controlling endogeneity issues. For example, Lu, Bao, Huang, Zhu, Mu, Chu, Xu, and Zha [110] provided a set of statistical strategies for reducing or eliminating endogeneity errors in panel data. To begin, he stated that the third-factor effect and control factors can be used to address endogeneity issues. Second, he claimed that predicted variable lags may be employed to solve the problem. Finally, he learned that the instrumental variable technique can help him solve this problem. Observable and unobservable effects in panel data can be managed using a lagged explanatory variable method. Fourth, he discovered that accounting for the unobservable heterogeneous effect in panel data with a fixed effect model is a smart idea. Finally, and most importantly, he supported the generalised method of moments (GMM) as a tool for dealing with endogeneity. Several other researchers have endorsed the GMM method for tackling this problem [121,142]. As a result, to appropriately evaluate this study, we used a fixed effect model and a GMM model.

### 3.4. Fixed Effect Model

The fixed effect model is an important tool for analyzing how independent and dependent variables within entities interact. Everything possesses unique characteristics that may or may not influence the expected variables, leading to incorrect outcomes. This problem has the potential to lead to erroneous results; therefore, it must be addressed. A fixed effect model is an important tool for resolving the problem in this scenario [7]. Panel data also have the disadvantage of being time-invariant, which can lead to biased or unfair results. As a result, the fixed effect tool is crucial in dealing with the time-invariant problem. The main problem is that panel data have unobservable heterogeneity [143].

The firm’s explanatory and predicted variables are strictly endogenous, implying that there is no relationship between them [142]. According to experts, the fixed effect tool is the most effective way for eliminating unobservable heterogeneity [7,139]. Using the fixed effect tool to remove time-invariant concerns and unobservable heterogeneity from panels has been demonstrated by some authors [144,145]. In econometric analysis, the fixed effect model is also known as a static panel model since it never enables the dependent variable’s lag to be replaced by the independent variable [142]. After this evaluation, the fixed effect model and the random effect model were utilized to analyze the data. The Hausman test value is used to evaluate whether a fixed effect or random effect model should be adopted [7,139].

### 3.5. Generalized Method of Moments (GMM)

The GMM methodology, also known as the dynamic panel model, was created by Arellano and Bond (1991) for panel data analysis. The cause-and-effect association between variables evolves through time. For empirical economics and finance studies using panel data, the GMM model is considered essential [115]. The GMM approach is regarded to be the most effective in resolving these issues when undertaking empirical research for exogenous or endogenous components. This method is also particularly well suited to obtaining trustworthy equation assessments [121].

In addition to linear and non-linear regressions, instrumental variables (IV), and the maximum likelihood approach, the GMM model has various categories. The majority of the studies used a fixed effect model or a first difference test to account for unobservable heterogeneity [144,145]. The GMM technique also includes the ability of the first difference test to cover unobservable variation in panel data [142,146]. In general, the GMM technique uses the lags of predicted variables. As a result, these variable lags are a powerful tool for dealing with panel data endogeneity [147].

The GMM model uses “internal modifying data” to deal with endogeneity [115]. Internal data altering is a statistical situation in which the prior value of a variable is subtracted from its present value. This concept is particularly useful for reducing the number of observations required and improving the proficiency of the GMM approach. The GMM model is the best technique for reducing endogeneity from panel data since it adds particular effects for changing coefficients [142,146]. Finally, the GMM model was employed to deal with panel data problems in order to achieve fair findings.

### 3.6. Feasible Generalized Least Square (FGLS)

When the residuals in a linear regression model have a high degree of correlation, feasible generalized least square (FGLS) is a method used for evaluating the unknown framework [148]. In 1934, Aitken [149] discovered FGLS for the first time. FGLS is the best strategy for dealing with heteroskedasticity. The ordinary least square (OLS) approach may become inefficient when the variance of the independent variables is not equal because the findings are confusing, causing estimators to make inaccurate assumptions.

The FGLS model is divided into two stages in the assessment process: when the regression equation is divided by a one-size deflator in the evaluation process to reduce biased results, and when the regression equation is divided by a one-size deflator in the evaluation process to minimize biased results [150]. Second, the erroneous terms in the equation are likely to be associated sequentially [151]. As a result, the probability of a sequential connection may be skewed. As a result, the FGLS model was used as a robustness technique to increase the results’ correctness in this study.

### 3.7. Econometric Equations

The following equations were created to investigate the role of green innovation strategy for corporate financing, with the moderating effects of CSR and management ownership [7,146].

(1) $$CF_{i,t}=\alpha_{1}+\beta_{1}GIS_{1i,t}+\gamma_{1}Z_{i,t}+\mu_{i,t}$$

(2) $$CF_{i,t}=\alpha_{2}+\beta_{2}CSR_{2i,t}+\gamma_{2}Z_{i,t}+\mu_{i,t}$$

(3) $$CF_{i,t}=\alpha_{3}+\beta_{3}GD_{3i,t}+\gamma_{3}Z_{i,t}+\mu_{i,t}$$

(4) $$CF_{i,t}=\alpha_{4}+\beta_{4}GIS_{4i,t}+\beta_{5}CSR_{5i,t}+\beta_{6}GIS*CSR_{6i,t}+\gamma_{4}Z_{i,t}+\mu_{i,t}$$

(5) $$CF_{i,t}=\alpha_{5}+\beta_{7}GIS_{7i,t}+\beta_{8}GD_{8i,t}+\beta_{9}GIS*GD_{9i,t}+\gamma_{5}Z_{i,t}+\mu_{i,t}$$

$CF_{i,t}$ reflects the corporate financing of firms i at year t, according to this equation: Green innovation strategy revealed by $GIS_{i,t}$: $CSR_{i,t}$ denotes a company’s social responsibility. $GD_{i,t}$ denotes gender diversity. $GIS*CSR_{i,t}$ represents the interplay between green innovative strategies and corporate social responsibility is depicted. $GIS*GD_{i,t}$ depicts the interplay between green innovative strategies and gender diversity firms i in the year t. Control variables of firm i at year t; $\mu_{i,t}$—error term; $\alpha_{n}$—constant term, *n* = 1; $\beta_{m},\;\gamma_{n}$. Estimated coefficients: *m* = 1, 2, 3, 4, 5, 6, 7, 8, 9.

## 4. Results and Discussion of the Investigation

### 4.1. Results

The descriptive statistics for corporate financing, green innovation strategy, corporate social responsibility, gender diversity, and control variables are presented in Table 1. This table displays the mean and standard deviation values. Table 1 also shows the results of the Pearson correlation test. Table 1 provides the Pearson coefficient correlation analysis results, which reveal the association between GIS and CF, with CSR and GD acting as moderators. The majority of the factors show a positive and substantial relationship. Likewise, all of the control variables show a positive and substantial relationship. This test has three levels of significance: one per cent, five per cent, and ten per cent.

The results of the fixed effect and GMM approach for the association between GIS and CF, CSR and CF, and GD and CF are shown in Table 2. To begin, model 1 shows that GIS has a significant and beneficial impact on CF, with fixed effect values (β_ = 0.338, *p* = 0.01) and GMM values (β_ = 0.340, *p* = 0.01). As a result, these results backed up the study’s initial premise, which stated that GIS is a useful tool for improving corporate financing. Second, model 2 shows that CSR has a significant and favorable impact on CF, with fixed effect values (β_ = 0.036, *p* = 0.01) and GMM values (β_ = 0.054, *p* = 0.01), respectively. As a result, these findings corroborated the study’s second hypothesis, which stated that corporate social responsibility can help the enhancement of corporate financing. Model 3 also demonstrates a link between GD and CF, with values from the fixed effect approach (β_ = 0.075, *p* = 0.01), and values from the GMM model (β_ = 0.092, *p* = 0.01). Finally, these findings are backed by the fourth hypothesis, according to which gender diversity can also be beneficial for corporate financing. Table 2 also shows the results of the Hausman test for models 1 through 3 (β_ = 774.82, *p* = 0.01; β_ = 60.77, *p* = 0.01; and β_ = 98.17, *p* = 0.01), confirming the fixed effect approach rather than the random effect method.

Table 3 shows the results of the link between the green innovation strategy and corporate financing, with the moderating effects of CSR and gender diversity. Model 4 illustrates the interaction values of green innovation strategy and corporate social responsibility (GIS*CSR) with fixed effect and GMM models (β_ = 0.580, *p* = 0.01; β_ = 0.425, *p* = 0.01). Finally, these data backed up hypothesis four, which indicated that corporate social responsibility can act as a moderator in the relationship between green innovation strategy and corporate financing. Model 5 also shows the interaction values of green innovation strategy and gender diversity (GIS ∗ GD) with fixed effect and GMM models, respectively (β_ = 0.018, *p* = 0.01; β_ = 0.022, *p* = 0.01). These findings also supported the study’s final hypothesis, which stated that gender diversity is an important mediator for increasing corporate financing using a green innovation strategy.

### 4.2. Additional Analysis

As a robustness test, this study ran an additional test to validate the results. This work used feasible generalized least squares (FGLS) to undertake extra analysis for the robustness test. Furthermore, heteroskedasticity emerges in a dataset when the standard errors of variables are observed over a period that is not constant [149]. Researchers claim that using FGLS can solve problems including autocorrelation and heteroskedasticity [151,152]. As a result, we use the FGLS model to solve these problems with the panel data.

Table 4 depicts the association between the green innovation strategy and corporate financing, the association between corporate social responsibility and corporate financing, the association between gender diversity and corporate financing, the moderating role of CSR on link green innovation strategy and corporate financing, and the moderating role of gender diversity on link green innovation strategy and corporate financing. Model 1 depicts GIS values with CF (β = 0.316, *p* = 0.01), model 2 depicts CSR with CF (β = 0.019, *p* = 0.01), model 3 depicts CSR with CF (β = 0.018, *p* = 0.01), model 4 depicts the GIS*CSR interaction (β = 0.067, *p* = 0.01), and model 5 depicts the GIS*GD interaction (β = 0.875, *p* = 0.01). As a result, all of these results from the FGLS approach corroborated the findings of all preceding procedures.

### 4.3. Discussion

The outcomes of this study back up the first hypothesis, which states that green innovation strategies boost corporate financing. In this support, prior several studies back up these assertions [12,13]. The financial support of major stakeholders might be credited for these results. So, the firm investment in patents and other green practices may enhance corporate financing. Furthermore, the theory of stakeholder justifies the findings of our study [21,40]. Firms in developing nations are motivated to engage in social practices to grow their operations in the international market [7]. These practices of firms entice more shareholders which may improve corporate financing activities. Firms that implement green practices, according to Porter (1991), generate innovation and gain market goodwill. According to Javeed and Lefen [153], company green activities aims are critical for corporate finance. Green innovation has the power to improve business value and future returns, and banks will give more loans since it meets the demands of green development proposals and green credit policies; therefore, general investors should increase their investments.

Furthermore, the outcomes of this research back up the second hypothesis, which states that corporate social responsibility is an effective tool for increasing corporate funding. In this context, prior several previous research studies, such as [26,27], have confirmed the favorable connection between corporate social responsibility and corporate financing. This finding shows the positive impact of CSR on company finance. According to the findings, adopting and implementing CSR methods that result in improved CSR performance leads to increased corporate finance for the organization. Better CSR performance reflects a company’s commitment to and engagement with stakeholders based on mutual trust and collaboration. In addition, the agency theory also supported these findings because managers will make a sequence of decisions to maximize their interests. In this scenario, for minimizing information asymmetry and agency concerns, CSR releases may serve as an observing tool [22].

Moreover, the findings support the third hypothesis which states that green innovation strategy and corporate funding have a positive relationship with CSR’s moderating effect. Prior research has found that companies who perform well in terms of CSR are more likely to publicly disclose their CSR initiatives by producing sustainability reports. These practices enhance shareholder confidence in firm green practices which will automatically provide a significant contribution to corporate financing. Theoretically, the stakeholder and agency theories both supported this link conceptually [21,22]. If firms and stakeholders maintain a positive relationship, they will be able to obtain the resources required for corporate financing [41]. CSR may not only help a firm deal with environmental issues, address public needs, and encourage social growth, but may also help improve financial decisions [28]. Furthermore, Cheng, Yang, and Sheu [27] demonstrated that CSR performance transparency influences financing decisions by lowering capital constraints, among other things. Firms that focus on CSR can boost their ability to innovate green practices [29]. Hence, our results supported the role of CSR as a moderator for association amid green innovative strategies and corporate financing.

Additionally, this study’s fourth hypothesis reported that gender diversity has a beneficial association with corporate finance. Our findings suggest that female executives are more connected with shareholders’ interests since they chose a higher proportion for corporate financing improvement. Because of their pervasive effect on the decision-making process, female directors can improve financing decisions and other developments [94]. A board of directors is the core of corporate governance. In China, about 70% of the listed companies have women on the board, which implies that their role in corporate governance should not be ignored. Additionally, the inclusion of women directors on the board decreases management opportunistic behavior and information asymmetry, which influences lenders’ estimates of the borrower’s capacity to return the debt with interest, and provides organizations with gender-diverse boards with a lower cost of debt [95]. In this context, the agency theory corroborated these findings in theory. A board with gender diversity, according to agency theory, is more likely to effectively monitor managers’ performance since diversity enhances financing decisions [39,49].

Lastly, these outcomes validate our fifth hypothesis, which states that green innovation strategy and corporate funding have a positive relationship with gender diversity as a moderating factor. Given that women’s environmental preferences may be stronger than men’s, gender-diverse boards are more likely to engage in environmental innovations than their industry peers. Furthermore, women on the board must hold at least two seats, firstly to have a positive impact on the firm’s environmental innovation, and secondly as increasing the representation of women on boards can increase the likelihood of green innovation. Our findings revealed that gender diversity can lead to the development of market green growth which will also improve corporate financing. Moreover, the agency theory relates to the role of gender diversity as a moderator in theory [22]. Additionally, Harjoto and Rossi [36] also reported that female directors assist firms to engage in green innovative methods for resolving agency conflicts. Many academics have underlined the value of gender diversity in improving green innovative operations and corporate finance [39,49,104]. The participation of female directors was also confirmed as a critical component of green innovation strategy and corporate financing [102,154].

## 5. Conclusions and Implications

### 5.1. Conclusions

Environmental concerns have garnered a lot of attention in developing nations, especially in the industrial sector, because this sector is more involved in generating environmental difficulties [83]. Additionally, strengthening the industrial sector is thought to be a key factor in the country’s economic success. As a result, this research demonstrates how corporate green initiatives can reduce industrial negative consequences while also improving company financing. The relationship between green innovation strategy and corporate funding is inspected in this study, with the role of corporate social responsibility and gender diversity as moderators. This study selects Chinese manufacturing enterprises from 2010 to 2019 for this aim.

This study first concludes that green innovation techniques help boost corporate financing after applying the fixed effect, the GMM model, and the FGLS. Companies that invest more in pollution management programmers generate more inventive goods and raise corporate funding because it improves their image and reputation in the eyes of stakeholders and society. Furthermore, corporate social responsibility is used as a moderator in this study. As a result, before using it as a moderator, this research looked at the direct impact of corporate social responsibility on corporate finance. As a result, the second finding of this study is that corporate social responsibility improves company funding by boosting shareholders’ interests.

Third, the outcomes of this study revealed that corporate social responsibility helps to increase the favorable association between green innovative strategy and corporate finance. Moreover, gender diversity is also used as a moderator in this research. Similarly, this study looked at the direct influence of gender diversity on corporate finance before utilizing it as a moderator. As a consequence, the study’s fourth finding is that gender diversity boosts corporate funding. Finally, the findings of this study show that gender diversity contributes to a more favorable relationship between green innovative strategy and corporate finance. Finally, this is the first study of its kind, which probed the link between green innovation strategy and corporate financing by using CSR and gender diversity as a moderator.

### 5.2. Policy Implications

Policymakers, governments, owners, firm management, and investors in both emerging and established nations will benefit from this research. To begin, businesses should implement green creative and social practices to eliminate or reduce environmental difficulties, which will improve the firm’s reputation in the eyes of the public. To enhance their performance, governments and stakeholders should put pressure on firms to embrace environmentally friendly practices and promote gender diversity. These findings show that CSR is a valuable instrument for environmental and social activities, as well as corporate funding. As a result, major shareholders should put pressure on management to improve business innovation by implementing CSR practices.

In addition, this study emphasized the relevance of gender diversity in improving sustainable firm practices. To increase corporate funding, every government and policymaker should encourage women to work in enterprises. Females are more likely to engage in social practices; thus, they should be represented on the board of directors and the sustainability committee. In addition, the study’s recommendation encourages company executives to engage in social practices. Green innovation strategies are regarded to be more socially responsible than those that do not. Because there has been little research on green innovative strategies and corporate funding, this study focuses on the role of green creative strategies in social development. Sustainable development also helps to alleviate environmental issues.

Businesses that engage in sustainable practices can see an instant rise in earnings and funding. Green practices and gender diversity on the board should be mandated by all regulatory institutions, particularly those in emerging economies. Furthermore, this study suggests that, as a form of moral support and encouragement, governments and institutions should honor enterprises with improved sustainable practices. Furthermore, social practices provide enterprises with long-term advantages, which implies that the cost of business social activities is less than the benefits. Moreover, firms from emerging economies may gain a positive reputation and image in the worldwide market after following sustainable practices. This study also encourages firms to stop engaging in unethical acts and engage in social practices.

According to the conclusions of this study, the industrial sector can play a critical role in environmental cleanup. International social authority and quality standards can also be useful in pressuring firms to conduct socially responsible behavior. Despite all the important implications, this study also has some limitations. Other components of corporate governance might be taken to look into green innovative techniques for business finance and profit. Gender diversity and CSR were used as moderators in this study; however, other essential aspects, such as CEOs, corporate performance, technology, market competitiveness, and so on, could be used as moderators and mediators in the future. This study is limited to Chinese market data; however, other emerging and developed countries may be explored in the future. This study focused on the industrial sector, although other areas could be explored in the future.

### 5.3. Limitation and Future Directions

This study found that major corporations are proactive in environmental practices, and small businesses should participate in these activities as well. So, the small firms can also be investigated in the future. Furthermore, this study focused on the manufacturing industry, but other industries may be investigated as well. Because of the data availability, only a short period was used for this investigation. This study advises that the function of business top management in proactive environmental initiatives and green innovation should be investigated in the future.

## Figures and Tables

**Table 1:** Descriptive statistics and Pearson correlation.

Variables	Mean	Standard Deviation	1	2	3	4	5	6	7	8	9	10	11
1. CF	0.307	0.386	1										
2. GIS	0.738	0.933	0.967 ***	1									
3. CSR	1.282	0.781	0.203 ***	0.224 ***	1								
4. GD	0.253	0.671	0.001	−0.041 **	−0.527 ***	1							
5. GISCSR	0.022	0.044	0.760 ***	0.675 ***	0.268 ***	−0.118 ***	1						
6. GISGD	0.024	0.110	0.251 ***	0.217 ***	−0.092 ***	0.039 ***	0.324 ***	1					
7. EA	0.428	0.434	−0.106 ***	−0.122 ***	0.409 ***	0.076 ***	−0.022 ***	−0.044 ***	1				
8. FS	8.080	6.084	0.134 ***	0.155 ***	0.516 ***	−0.022 ***	0.104 ***	−0.065 ***	0.425 ***	1			
9. ATO	6.363	5.144	0.233 ***	0.255 ***	0.135 ***	-0.243 ***	0.196 ***	0.067 ***	−0.243 ***	−0.049 ***	1		
10. PPE	0.501	0.524	0.702 ***	0.669 ***	0.201 ***	0.015 ***	0.641 ***	0.226 ***	0.172 ***	0.095 ***	0.120 ***	1	
11. LIQ	0.222	0.391	0.542 ***	0.486 ***	0.178 ***	0.083 ***	0.455 ***	0.147 ***	0.232 ***	0.466 ***	0.003 ***	0.678 ***	1

Significance levels: ** *p* < 0.05, *** *p* < 0.01. CF reveals corporate financing; GIS shows the green innovation strategy; CSR presents corporate social responsibility; GD reveals gender diversity; FS shows the firm size; LIQ reveals the liquidity; PPE highlights the ratio of plant, property, and equipment; ATO shows the asset turnover; and EA reveals environmental awareness.

**Table 2:** Results of the link between GIS and CF, CSR and CF, GD and CF.

	Model 1		Model 2		Model 3	
Variables	Corporate Financing		Corporate Financing		Corporate Financing	
	Fixed Effect	GMM	Fixed Effect	GMM	Fixed Effect	GMM
GIS	0.338 ***	0.340 ***				
CSR			0.036 ***	0.054 ***		
GD					0.075 ***	0.092 ***
EA	−0.101 ***	−0.130 ***	−0.469 ***	−0.617 ***	−0.466 ***	−0.615 ***
FS	0.106 ***	−0.119 ***	0.091	0.352 ***	0.137	0.447 ***
ATO	0.003 ***	0.002 ***	0.007 ***	0.004 ***	0.008 ***	0.004 ***
PPE	0.118 ***	0.115 ***	0.548 ***	0.579 ***	0.547 ***	0.578 ***
LIQ	−0.016 ***	−0.014 ***	0.023	−0.008 ***	0.019 ***	−0.012
Constant	0.021 ***	0.023 ***	0.131 ***	0.116 ***	0.154 ***	0.154 ***
R^2^	0.9711		0.6653		0.6663	
F	20.80 ***		12.02 ***		12.37	
N	3006	2403	3006	2403	3006	2403
Hausman Test	774.82 ***		60.77 ***		98.17 ***	
Wald Chi^2^		89,566.40 ***		4168.49 ***		4167.28 ***

Significance levels: *** *p* < 0.01.

**Table 3:** The moderating results.

	Model 4		Model 5	
Variables	Corporate Financing		Corporate Financing	
	Fixed Effect	GMM	Fixed Effect	GMM
GIS	0.327 ***	0.330 ***	0.331 ***	0.340 ***
CSR	-0.002	0.008		
GISCSR	0.580 ***	0.425 ***		
GD			0.018 ***	0.022 ***
GISGD			0.044 ***	0.026 ***
EA	−0.093 ***	−0.121 ***	−0.101 ***	−0.128 ***
FS	0.113 ***	−0.104 ***	0.109 ***	0.118 ***
ATO	0.002 ***	0.001 ***	0.007 ***	0.002 ***
PPE	0.108 ***	0.106 ***	0.117 ***	0.113 ***
LIQ	−0.015 ***	−0.013 ***	−0.016 ***	−0.014 ***
Constant	0.024***	0.012 ***	0.015 ***	0.017 **
R^2^	0.9743		0.9615	
F	15.79 ***		19.92 ***	
N	3006	2403	3006	2403
Hausman Test	564.03 ***		433.66 ***	
Wald Chi^2^		98,063.04 ***		90,936.28 ***

Significance levels: ** *p* < 0.05, *** *p* < 0.01.

**Table 4:** The robustness results with FGLS.

	Model 1	Model 2	Model 3	Model 4	Model 5
Variables	Corporate Financing		Corporate Financing		Corporate Financing
	FGLS	FGLS	FGLS	FGLS	FGLS
GIS	0.316 ***			0.281 ***	0.313 ***
CSR		0.019 ***		−0.011 ***	
GISCSR				2.279 ***	
GD			0.018 ***		0.014 ***
GISGD					0.067 ***
EA	−0.037 ***	−0.249 ***	−0.237 ***	−0.024 ***	−0.044 ***
FS	0.010	0.624 ***	0.689 ***	0.070 ***	0.027 **
ATO	−0.001 ***	0.002 ***	0.004 ***	−0.005 ***	0.001 **
PPE	0.159 ***	0.586 ***	0.582***	0.107 ***	0.158 ***
LIQ	0.025 ***	0.026 **	0.023 ***	−0.008 **	0.021 ***
Constant	0.003 ***	0.014 ***	0.013 ***	0.015 ***	−0.001 ***
N	3006	3006	3006	3006	3006
Wald Chi^2^	147,243.45 ***	22,324.66 ***	29,497.17 ***	248,769.99 ***	131,969.86 ***

Significance levels: ** *p* < 0.05, *** *p* < 0.01.

## Data Availability

Not applicable.
